# Screening Familial Risk for Hereditary Breast and Ovarian Cancer

**DOI:** 10.1001/jamanetworkopen.2024.35901

**Published:** 2024-09-25

**Authors:** Daniel Kiser, Gai Elhanan, Alexandre Bolze, Iva Neveux, Karen A. Schlauch, William J. Metcalf, Elizabeth T. Cirulli, Catherine McCarthy, Leslie A. Greenberg, Savanna Grime, Jamie M. Schnell Blitstein, William Plauth, Joseph J. Grzymski

**Affiliations:** 1 University of Nevada Reno School of Medicine, Reno; 2 Helix, Inc, San Mateo, California; 3 Renown Health, Reno, Nevada

## Abstract

**Importance:**

Most patients with pathogenic or likely pathogenic (P/LP) variants for breast cancer have not undergone genetic testing.

**Objective:**

To identify patients meeting family history criteria for genetic testing in the electronic health record (EHR).

**Design, Setting, and Participants:**

This study included both cross-sectional (observation date, February 1, 2024) and retrospective cohort (observation period, January 1, 2018, to February 1, 2024) analyses. Participants included patients aged 18 to 79 years enrolled in Renown Health, a large health system in Northern Nevada. Genotype was known for 38 003 patients enrolled in Healthy Nevada Project (HNP), a population genomics study.

**Exposure:**

An EHR indicating that a patient is positive for criteria according to the Seven-Question Family History Questionnaire (hereafter, FHS7 positive) assessing familial risk for hereditary breast and ovarian cancer (HBOC).

**Main Outcomes and Measures:**

The primary outcomes were the presence of P/LP variants in the *ATM*, *BRCA1*, *BRCA2*, *CHEK2*, or *PALB2* genes (cross-sectional analysis) or a diagnosis of cancer (cohort analysis). Age-adjusted cancer incidence rates per 100 000 patients per year were calculated using the 2020 US population as the standard. Hazard ratios (HRs) for cancer attributable to FHS7-positive status were estimated using cause-specific hazard models.

**Results:**

Among 835 727 patients, 423 393 (50.7%) were female and 29 913 (3.6%) were FHS7 positive. Among those who were FHS7 positive, 24 535 (82.0%) had no evidence of prior genetic testing for HBOC in their EHR. Being FHS7 positive was associated with increased prevalence of P/LP variants in *BRCA1*/*BRCA2* (odds ratio [OR], 3.34; 95% CI, 2.48-4.47), *CHEK2* (OR, 1.62; 95% CI, 1.05-2.43), and *PALB2* (OR, 2.84; 95% CI, 1.23-6.16) among HNP female individuals, and in *BRCA1*/*BRCA2* (OR, 3.35; 95% CI, 1.93-5.56) among HNP male individuals. Being FHS7 positive was also associated with significantly increased risk of cancer among 131 622 non-HNP female individuals (HR, 1.44; 95% CI, 1.22-1.70) but not among 114 982 non-HNP male individuals (HR, 1.11; 95% CI, 0.87-1.42). Among 1527 HNP survey respondents, 352 of 383 EHR-FHS7 positive patients (91.9%) were survey-FHS7 positive, but only 352 of 883 survey-FHS7 positive patients (39.9%) were EHR-FHS7 positive. Of the 29 913 FHS7-positive patients, 19 764 (66.1%) were identified only after parsing free-text family history comments. Socioeconomic differences were also observed between EHR-FHS7-negative and EHR-FHS7-positive patients, suggesting disparities in recording family history.

**Conclusions and Relevance:**

In this cross-sectional study, EHR-derived FHS7 identified thousands of patients with familial risk for breast cancer, indicating a substantial gap in genetic testing. However, limitations in EHR family history data suggested that other identification methods, such as direct-to-patient questionnaires, are required to fully address this gap.

## Key Points

**Question:**

In a large health system, how many ungenotyped patients meet family history genetic testing criteria for hereditary breast and ovarian cancer?

**Findings:**

In this cross-sectional analysis, 2.9% of patients had no evidence of prior genetic testing but had electronic health records indicating they met family history criteria. These criteria were associated with significantly increased prevalence of genetic risk variants among 38 003 genotyped patients.

**Meaning:**

These findings suggest that substantial gaps exist in identifying and testing patients meeting family history criteria for hereditary breast and ovarian cancer, and other methods may be needed to close these gaps.

## Teaser

This cross-sectional study examines whether patients meeting the family history criteria for genetic testing for breast cancer can be identified through electronic health records.

## Introduction

Evidence suggests that up to 80% of patients with a pathogenic or likely pathogenic (P/LP) variant in the *BRCA1* and *BRCA2* (*BRCA1/2*) genes are unaware of their condition and subsequent risk for hereditary breast and ovarian cancer (HBOC).^1,2,3,4,5^ Rare or less penetrant P/LP variants in genes such as *PALB2*, *CHEK2*, and *ATM* are even less likely to be detected.^6^ This is concerning, because the lifetime risk for breast cancer exceeds 50% for women with P/LP variants in *BRCA1/2* and 20% for women with P/LP variants in *PALB2*, *CHEK2*, and *ATM* (compared with a baseline risk of 12%).^7,8,9^ Men are also affected by these variants, with increased risk of breast, prostate, and other cancers.^10,11,12,13,14,15,16^

This combination of high risk and low awareness can be addressed by early and population-wide genetic testing, enabling preventive treatment and earlier cancer detection. As of January 2024, the Healthy Nevada Project (HNP)^1,17,18^ had notified 330 individuals with P/LP variants in *BRCA1* or *BRCA2* genes (*BRCA1/2* positive) of their genetic risk, of a study population of more than 53 000 patients. However, current HNP participants compose less than 7% of the 800 000 patients who have had clinical encounters at the health system sponsoring the HNP. Given an expected prevalence of *BRCA1/2 *positivity of approximately 1 in 200 individuals^1,2^ to 1 in 400 individuals,^19^ depending on the reference population, it is likely that there are at least an additional 1000 patients in the health system who are unaware of their genetic risk.

There is growing concern in our community about this gap in genetic testing, as indicated by recent Nevada legislation (Senate Bill 251 [SB251])^20^ requiring counseling and payer coverage of *BRCA1/2* testing for women who meet US Preventive Services Task Force (USPSTF) guidelines.^21^ The USPSTF guidelines recommend that women with either a personal or family history of certain cancers be assessed with one of several simple family history tools, such as the Seven-Question Family History Questionnaire (FHS7),^22^ to determine whether they should be referred for genetic services.

Our analysis was motivated by SB251 and our prior work^4^ seeking to improve clinical follow-up for *BRCA1/2*-positive patients. Thus, the goal in this study was to use FHS7 to identify patients who meet USPSTF family history criteria in their electronic health record (EHR) but who have not undergone genetic testing for HBOC. We also evaluated the utility of EHR-derived FHS7 by assessing its association with genetic risk variants and cancer incidence. On the basis of our results, we propose scalable strategies that can be used within a health care system to substantially increase the proportion of at-risk patients who undergo testing.

## Methods

All human participant research for the HNP is institutional review board (IRB) approved. Both the cross-sectional and cohort analyses were performed using health system EHR data with personal identifiers removed, and the study was deemed IRB exempt by the University of Nevada, Reno IRB as part of a larger body of work. All HNP participants provided written, informed consent. This report followed Strengthening the Reporting of Observational Studies in Epidemiology (STROBE) reporting guidelines for observational studies.^23^

### Data

We used a cross-sectional study design and a retrospective cohort study design to assess the association of EHR-derived FHS7 status with P/LP variants and cancer, respectively. Included patients were aged 18 to 79 years, were not deceased, and had at least 1 personal history or family history assessment in their record at the start of the analysis periods. EHR data were used to obtain demographic and health care utilization variables; current and personal history diagnoses of HBOC-related cancers, which we defined as melanoma and breast, ovarian, fallopian tube, prostate, pancreatic, colorectal, and peritoneal cancer; evidence of prior genetic testing for HBOC, based on dedicated local system diagnosis codes as described in Elhanan et al^4^; and dates of death. Additional dates of death were obtained from death certificates,^24^ and median household income for patient Census tracts of residence was obtained from the US Census Bureau.^25^ All diagnoses were retrieved using local system diagnostic codes, most of which were mapped to *International Statistical Classification of Diseases, Tenth Revision, Clinical Modification* codes. Race and ethnicity data were extracted from the EHR primarily to assess potential biases in family history collection, with race and ethnicity options being defined by the health system but chosen by the patients. Because some race and ethnicity definitions were overlapping, races were grouped into the categories American Indian or Alaska Native, Asian, Black or African American, Native Hawaiian or Pacific Islander, White, multiracial (if >1 race was selected), and other, which includes Hispanic or Latino, Middle Eastern/North African, or any other race or ethnicity not otherwise specified. Indications of Hispanic or Latino race were combined with indications of Hispanic ethnicity (eg, Cuban, Hispanic Origin, Mexican, or Puerto Rican) to define Hispanic ethnicity.

Family history of HBOC-related cancers was extracted from the EHR using a combination of discrete codes and string matches in the comment field (eMethods in Supplement 1). Duplicate records and records with negations, terms indicating uncertainty, nonblood relatives, and gender-conflicted conditions were removed. To determine USPSTF eligibility for genetic services, we used the extracted records to assess whether patients meet criteria in the FHS7 questions (Table 1). Meeting just 1 of the 7 criteria in FHS7 indicates possible familial risk.

**Table 1.** FHS7 Questions and Number of Patients Who Met FHS7 Criteria^a^

Variable	Patients, No. (%)	
	Survey FHS7 positive (n = 1527)^b^	EHR-derived FHS7 positive (n = 835 727)^c^
FHS7 positive	883 (57.8)	29 913 (3.6)
Distribution of patients meeting criteria, No. (% of FHS7 positive)		
Question 1. Did any woman in your family have breast cancer before the age of 50 y?^d^	428 (48.5)	2338 (7.8)
Question 2. Did any man in your family have breast cancer?	11 (1.2)	640 (2.1)
Question 3. Did any of your relatives have bilateral breast cancer?	67 (7.6)	239 (0.8)
Question 4. Did any woman in your family have breast and ovarian cancer?	172 (19.5)	701 (2.3)
Question 5. Did any of your first degree relatives have breast or ovarian cancer?	141 (16.0)	22 896 (76.5)
Question 6. Do you have 2 or more relatives with breast and/or ovarian cancer?	23 (2.6)	8170 (27.3)
Question 7. Do you have 2 or more relatives with breast and/or bowel cancer?	42 (4.8)	11 346 (37.9)

Abbreviations: EHR, electronic health record; FHS7, Seven-Question Family History Questionnaire.

^a^ Meeting 1 of the 7 criteria is sufficient to indicate referral for genetic screening.

^b^ Survey ended with the first affirmative response, with questions being ordered 1 through 7.

^c^ Percentages of responses sum to greater than 100% since participants can meet criteria in more than one question.

^d^ For this study, relatives of unknown sex (ie, *sibling*) were grouped together with women when assessing this question, since it would also result in FHS7-positive status if they were male (question 2).

For participants who joined HNP by October 2023, P/LP variants in *ATM*, *BRCA1*, *BRCA2*, *CHEK2*, and *PALB2* were identified on the basis of loss-of-function prediction in the MANE (Matched Annotation from National Center for Biotechnology Information and European Molecular Biology Laboratory–European Bioinformatics Institute)^26^ transcript and ClinVar expert panel interpretations^9^ or on the basis of previous notification by HNP of Clinical Laboratory Improvement Amendments–certified laboratory results. HNP participants who provide consent are invited to complete a health determinants survey, to which the FHS7 questions were added in March 2023. Between March 2023 and January 2024, 7767 HNP participants were invited to complete the updated survey. Participants answering yes to at least 1 of the FHS7 questions were defined as FHS7 positive.

### Cross-Sectional Analysis

To compare EHR-derived FHS7 status with FHS7 responses in the survey and to assess the association of EHR-derived FHS7 status with genetic risk variants, we used EHR data available February 1, 2024. We also assessed whether including a personal history of breast, ovarian, fallopian tubal, peritoneal, or prostate cancer (cancers mentioned in National Comprehensive Cancer Network guidelines^27^) as an additional criterion to EHR-derived FHS7 improved accuracy for identifying genetic risk variants.

### Cohort Analysis

Because HNP participants may not be representative of the overall health system population, we also examined the risk associated with being FHS7 positive among patients not enrolled in HNP (hereafter, non-HNP patients). Because of the lack of genetic data for these patients, we used incidence of cancer as an outcome instead of the presence of P/LP variants, retrospectively following FHS7-positive and FHS7-negative cohorts from January 1, 2018, to February 1, 2024. Cohorts were separated by biological sex: female cancer types were breast, ovarian, and fallopian tubal; and male cancer types were breast, prostate, and pancreatic. To reduce the possible effect that suspicion of cancer might have on the recording of relevant family history, FHS7 status was determined 180 days before the start date of observation. We also excluded patients whose first diagnosis indicated a history of cancer, since in those cases the age of onset cannot be determined.

### Statistical Analysis

All analyses were conducted using R statistical software version 4.4.0 (R Project for Statistical Computing). In the cohort analysis, we calculated age-adjusted cancer incidence rates per 100 000 patients per year, using the 2020 US population as the standard.^28^ Hazard ratios (HRs) attributable to FHS7-positive status were estimated using cause-specific hazard models^29^ with stratification by cancer history, sociodemographic characteristics, and health care utilization levels (eTable 1 in Supplement 1). Statistical significance was defined as a 95% CI that did not contain the reference value (1 for ORs and HRs and $0 for the median of the difference in monetization). Observations were censored at date of death or the last date on which a patient was charged for services by the health system. To generate and implement the hazard models, we used the R package survival, and Schoenfeld residuals^30^ were assessed to check for violations of the proportional hazards assumption. In sex-specific analyses, we excluded nonbinary patients or those with unknown sex because the numbers were too low to yield informative results.

## Results

### Cross-Sectional Analysis

Among our cross-sectional analysis population of 835 727 patients, 457 548 (54.7%) were younger than 50 years and 423 393 (50.7%) were female (Table 2). A family history of at least 1 relative with an HBOC-related cancer was found in the EHRs of 73 666 patients (8.8%), and 29 913 (3.6%) were FHS7 positive. A total of 102 853 relatives with HBOC-related cancers were identified, but 72 598 (70.6%) of these relatives and 19 764 of 29 913 FHS7-positive patients (66.1%) would have remained unidentified if free-text comments had been ignored; 93 178 relatives (90.6%) were recorded by practitioners, who used free-text comments at a higher rate than patients (in 67 905 records [72.9%] vs 4690 records [48.5%]). The most common free-text comments are listed in eTable 2 in Supplement 1. The age of onset for cancers was recorded discretely for 15 397 relatives (15.0%). When computing EHR-derived FHS7 status, the fifth and seventh FHS7 questions yielded positive results most frequently (Table 1). Among the 29 913 FHS7-positive patients, 24 535 (82.0%) had no EHR evidence of genetic testing results for HBOC and were not HNP participants (Table 2), indicating that they had likely not been assessed for genetic risk.

**Table 2.** Cross-Sectional Study Population Demographics and Other Characteristics

Characteristic	Patients, No. (%)		
	Patients with personal and/or family history assessment (n = 835 727)	FHS7-positive patients (n = 29 913)	HNP participants (n = 38 003)
Aged <50 y	457 441 (54.7)	10 411 (34.8)	16 937 (44.6)
Sex			
Female	423 393 (50.7)	21 780 (72.8)	26 297 (69.2)
Male	411 986 (49.3)	8128 (27.2)	11 699 (30.8)
Unknown or other^a^	348 (<0.1)	5 (<0.1)	7 (<0.1)
Known race	792 848 (94.9)	29 090 (97.2)	37 310 (98.2)
Race, No. (% of known race)			
American Indian or Alaska Native	8593 (1.1)	185 (0.6)	300 (0.8)
Asian	29 428 (3.7)	929 (3.2)	1365 (3.7)
Black or African American	25 826 (3.3)	571 (2.0)	707 (1.9)
Native Hawaiian or Pacific Islander	4264 (0.5)	115 (0.4)	137 (0.4)
White	645 979 (81.5)	25 921 (89.1)	32 649 (87.5)
Multiracial	7601 (1.0)	303 (1.0)	546 (1.5)
Other^b^	71 157 (9.0)	1066 (3.7)	1606 (4.3)
Hispanic ethnicity	127 890 (15.3)	2526 (8.4)	4240 (11.2)
Non-English speaking	28 502 (3.4)	252 (0.8)	120 (0.3)
Known US Census tract	708 905 (84.8)	25 903 (86.6)	34 480 (90.7)
Median household income of US Census tract of residence, No. (% of known US Census tract)			
<$50 000	141 255 (19.9)	3300 (12.7)	4515 (13.1)
$50 000-99 999	423 987 (59.8)	16 004 (61.8)	20 826 (60.4)
≥$100 000	143 663 (20.3)	6599 (25.5)	9139 (26.5)
Patients with charges	684 093 (81.9)	28 394 (94.9)	37 583 (98.9)
Health insurance category, No. (% of patients with charges)			
Private (no Medicaid)	405 918 (59.3)	20 456 (72.0)	28 451 (75.7)
Medicare (no private or Medicaid)	56 910 (8.3)	3008 (10.6)	3053 (8.1)
Medicaid (ever used)	135 398 (19.8)	4101 (14.4)	5060 (13.5)
Self-pay only	43 337 (6.3)	209 (0.7)	351 (0.9)
Other^c^	42 530 (6.2)	620 (2.2)	668 (1.8)
Health care utilization, median (IQR)			
Personal history/family history assessments	4 (1-12)	17 (8-33)	17 (8-34)
Monetization, $1000^d^	4.9 (0.4-30.7)	26.2 (5.7-82.6)	34.0 (9.5-89.3)
Personal history of cancer			
Any cancer	29 198 (3.5)	3473 (11.6)	2974 (7.8)
Breast	11 612 (1.4)	2065 (6.9)	1389 (3.7)
Colorectal	3614 (0.4)	338 (1.1)	292 (0.8)
Melanoma	6248 (0.7)	702 (2.3)	783 (2.1)
Ovarian	1427 (0.2)	194 (0.6)	147 (0.4)
Pancreatic	670 (0.1)	47 (0.2)	52 (0.1)
Peritoneal	104 (<0.1)	8 (<0.1)	5 (<0.1)
Prostate	7257 (0.9)	398 (1.3)	530 (1.4)
Fallopian tubal	79 (<0.1)	7 (<0.1)	12 (<0.1)
Prior knowledge of HBOC			
Diagnosis	1674 (0.2)	623 (2.1)	413 (1.1)
HNP participant	38 003 (4.5)	4942 (16.5)	38 003 (100.0)
No diagnosis or non-HNP	796 463 (95.3)	24 535 (82.0)	0
Family history records			
HBOC-related family history	73 666 (8.8)	29 913 (100.0)	10 650 (28.0)
FHS7 positive	29 913 (3.6)	29 913 (100.0)	4942 (13.0)

Abbreviations: FHS7, Seven-Question Family History Questionnaire; HBOC, hereditary breast and ovarian cancer; HNP, Healthy Nevada Project.

^a^ Includes sex listed as X or nonbinary.

^b^ Includes races listed in the electronic health record as Hispanic or Latino, Middle Eastern/North African, or any other race not otherwise specified.

^c^ Refers to financial classes recorded as accident liability, Blue Shield, corporate, county, group health plan, negotiated discount, other government payers, special programs, Tricare, worker’s compensation, or other.

^d^ Refers to charges to a patient’s account.

Compared with the overall study population, FHS7-positive patients were less likely to be younger than 50 years (odds ratio [OR], 0.43; 95% CI, 0.42-0.44), to report Hispanic ethnicity (OR, 0.50; 95% CI, 0.48-0.52), and to live in a low-income Census tract (OR, 0.60; 95% CI, 0.58-0.62), and they were more likely to be female (OR, 2.70; 95% CI, 2.63-2.77) and of White race (OR, 1.95; 95% CI, 1.88-2.01). Healthcare utilization was higher for FHS7-positive patients, with the median of the difference in monetization between FHS7-positive and FHS7-negative patients being $12 105 (95% CI, $11 757-$12 459). FHS7-positive patients also had a higher rate of personal history of cancer than in the overall study population (OR, 3.98; 95% CI, 3.84-4.14). However, for 3719 instances (among 3675 patients) in which a particular cancer type appeared as both a personal history diagnosis and in the family history, family history was recorded on the same day as the first diagnosis 446 times (12.0%) and after the first diagnosis 1866 times (50.2%).

In our cross-sectional population, 38 003 patients (4.5% of the total) were HNP participants (Table 2). Among the 7767 HNP participants who were invited to complete the updated health determinants survey, 1690 (21.8%) responded to the FHS7 questions. Among respondents, 1635 (96.7%) were health system patients, and 1527 (90.4%) were among the 835 727 patients in our study population. Of the 1527 study participants, 883 (57.8%) were FHS7 positive on the basis of their survey responses. The distribution of affirmative responses is shown in Table 1. As with EHR-FHS7, we observed a sex bias in survey responses: male individuals were FHS7 positive in the survey at a lower rate (150 of 345 individuals [43.5%]) than female individuals (733 of 1182 individuals [62.0%]). Of 383 patient respondents who were FHS7 positive in their EHR, 352 (91.9%) were also FHS7 positive in the survey; however, these 352 patients were only 39.9% of the 883 who were FHS7 positive in the survey. Thus, survey results suggest that most patients who are FHS7 positive in their EHR truly meet family history criteria, but that EHR-derived FHS7 may miss many patients who would be FHS7 positive if approached with a direct questionnaire.

We then assessed whether EHR-derived FHS7-positive status was indeed associated with an increased risk of carrying a P/LP in one of the cancer genes. Both male (OR, 3.35; 95% CI, 1.93-5.56) and female (OR, 3.34; 95% CI, 2.48-4.47) FHS7-positive HNP participants were 3 times more likely to be *BRCA1/2* positive, and female individuals were 1.62 (95% CI, 1.05-2.43) and 2.84 (95% CI, 1.23-6.16) times more likely to have P/LP variants in *CHEK2* or *PALB2*, respectively (Table 3 and Table 4). The number needed to test (NNT) to detect 1 *BRCA1/2*-positive patient decreased from 128 to 53 for female individuals and from 119 to 42 for male individuals when prescreening with FHS7. However, sensitivity (the proportion of patients with P/LP variants who were successfully identified) was only 0.20 for *BRCA1/2-*positive male individuals, compared with 0.38 for *BRCA1/2*-positive female individuals, and incorporating a personal history of cancer as an inclusion criterion reduced NNT for female individuals but not for male individuals.

**Table 3.** Accuracy of FHS7 and Personal History of Cancer for Identifying P/LP Variants in Female Individuals Enrolled in the Health Nevada Project

Variant	Total individuals (n = 26 297)		FHS7 positive (n = 4107)					FHS7 positive or personal history of cancer (n = 5189)^a^				
	No.^b^	NNT^c^	No.	NNT^c^	OR (95% CI)^d^	Sensitivity	Specificity	No.	NNT^c^	OR (95% CI)^d^	Sensitivity	Specificity
Any monogenic P/LP variant	458	58	131	32	2.20 (1.78-2.71)	0.29	0.85	201	26	3.27 (2.70-3.96)	0.44	0.81
*ATM*	81	325	10	411	0.76 (0.35-1.48)	0.12	0.84	22	236	1.52 (0.89-2.52)	0.27	0.80
*BRCA1*	87	303	36	115	3.84 (2.43-6.01)	0.41	0.84	56	93	7.42 (4.70-11.92)	0.64	0.80
*BRCA2*	120	220	42	98	2.93 (1.96-4.32)	0.35	0.84	61	86	4.24 (2.91-6.18)	0.51	0.80
*BRCA1/2*	206	128	78	53	3.34 (2.48-4.47)	0.38	0.85	116	45	5.34 (4.01-7.13)	0.56	0.81
*CHEK2*	139	190	32	129	1.62 (1.05-2.43)	0.23	0.84	48	109	2.16 (1.49-3.10)	0.35	0.80
*PALB2*	32	822	11	374	2.84 (1.23-6.16)	0.34	0.84	15	346	3.60 (1.67-7.66)	0.47	0.80

Abbreviations: FHS7, Seven-Question Family History Questionnaire; NNT, number needed to treat; OR, odds ratio; P/LP, pathogenic or likely pathogenic.

ᵃRefers to breast, ovarian, fallopian tubal, peritoneal, or pancreatic cancer.

ᵇOne patient had P/LP variants in both *BRCA1* and *BRCA2*.

ᶜRefers to NNT to detect 1 patient with a P/LP variant.

ᵈReference is not meeting FHS7 or personal history screening criteria.

**Table 4.** Accuracy of FHS7 and Personal History of Cancer for Identifying P/LP Variants in Male Individuals Enrolled in the Health Nevada Project

Variant	Total individuals (n = 11 699)		FHS7 positive (n = 835)					FHS7 positive or personal history of cancer (n = 1352)^a^				
	No.^b^	NNT^c^	No.	NNT^c^	OR (95% CI)^d^	Sensitivity	Specificity	No.	NNT^c^	OR (95% CI)^d^	Sensitivity	Specificity
Any monogenic P/LP variant	217	54	31	27	2.21 (1.45-3.28)	0.14	0.93	45	31	2.04 (1.43-2.86)	0.21	0.89
*ATM*	33	355	4	209	1.80 (0.46-5.14)	0.12	0.93	4	338	1.06 (0.27-3.01)	0.12	0.88
*BRCA1*	35	335	9	93	4.54 (1.87-10.04)	0.26	0.93	11	123	3.53 (1.56-7.50)	0.31	0.89
*BRCA2*	64	183	11	76	2.72 (1.28-5.30)	0.17	0.93	14	97	2.15 (1.10-3.97)	0.22	0.89
*BRCA1/2*	99	119	20	42	3.35 (1.93-5.56)	0.20	0.93	25	55	2.62 (1.59-4.18)	0.25	0.89
*CHEK2*	66	178	5	167	1.07 (0.33-2.64)	0.08	0.93	13	104	1.89 (0.94-3.52)	0.20	0.88
*PALB2*	21	558	2	418	1.37 (0.15-5.70)	0.10	0.93	3	451	1.28 (0.24-4.38)	0.14	0.88

Abbreviations: FHS7, Seven-Question Family History Questionnaire; NNT, number needed to treat; OR, odds ratio; P/LP, pathogenic or likely pathogenic.

ᵃRefers to breast, peritoneal, pancreatic, or prostate cancer.

ᵇTwo patients had multiple P/LP variants (*ATM/BRCA2* and *BRCA1/PALB2*).

ᶜRefers to NNT to detect 1 patient with a P/LP variant.

ᵈReference is not meeting FHS7 or personal history screening criteria.

### Cohort Analysis

After exclusions, we had 131 622 female individuals (6134 [4.7%] FHS7 positive) and 114 982 male individuals (2337 [2.0%] FHS7 positive) in our non-HNP study cohorts (eTable 3 in Supplement 1) used for assessing the association of FHS7-positive status with the incidence of breast, ovarian, and fallopian tubal cancer in female individuals and breast, prostate, and pancreatic cancer in male individuals. For female individuals, total observation time was 5.8 × 10^5^ years, for a mean (SD) of 4.4 (1.8) years per patient; for male individuals, total observation time was 4.8 × 10^5^ years, for a mean (SD) of 4.2 (1.8) years per patient. As in the cross-sectional study population, substantial socioeconomic and health care utilization differences were observed between FHS7-negative and FHS7-positive cohorts. During the observation period, 2085 female individuals (1.6% overall) and 2181 male individuals (1.9% overall) developed cancer. Age-adjusted cancer incidence rates were 239.3 cases per 100 000 per year for FHS7-negative female individuals, 367.2 cases per 100 000 per year for FHS7-positive female individuals, 260.1 cases per 100 000 per year for FHS7-negative male individuals, and 309.9 cases per 100 000 per year for FHS7-positive male individuals. For FHS7-positive vs FHS7-negative patients, the cause-specific hazard models yielded HRs for receiving a diagnosis of cancer of 1.44 (95% CI, 1.22-1.70) among female individuals and 1.11 (95% CI, 0.87-1.42) among male individuals.

## Discussion

Programs like HNP,^1^ MyCode,^2^ and BioMe^3^ have demonstrated the value of population-wide genetic testing for reducing the large number of individuals with undetected P/LP variants in *BRCA1/2* and other Centers for Disease Control and Prevention Tier 1 genes (ie, genes in which P/LP variants have been identified as being highly penetrant but for which effective interventions exist).^31^ At least 1 insurer plans to begin covering such testing,^32^ although there are still some uncertainties about appropriate follow-up for positive findings^4^ and potential for harm.^33,34,35^ However, the benefits of testing at-risk populations are better established, as indicated by American Society of Breast Surgeons recommendations to test anyone with a personal history of breast cancer,^36^ and Nevada’s SB251 requirements that payers cover testing for women who meet familial risk criteria.^20^ Our cross-sectional examination of existing EHR family history data at our health system revealed 24 535 patients who are at risk according to FHS7 but have no EHR evidence of testing. Also, survey results and the FHS7 validation conducted by Ashton-Prolla et al^22^ suggest that the 3.6% FHS7-positive rate we observed in our health system is an underestimate of the true incidence, and that a direct questionnaire, while still identifying more than 90% of patients who meet family history criteria in their EHR, would identify many additional at-risk patients. This indicates substantial gaps in risk assessment and testing. In this discussion, we advocate for closing these gaps using risk assessment processes that are low-cost, sustainable, based on reliable data, and equitable.

Compared with population-wide genetic testing, prescreening with tools like FHS7 can reduce costs by reducing the NNT: among HNP participants, the NNT to detect 1 *BRCA1/2*-positive patient decreased from 128 to 53 for female individuals and from 119 to 42 for male individuals when prescreening with FHS7. NNT for female individuals decreased further when a personal history of cancer was included as a criterion. Costs can also be reduced by bypassing genetic counseling before testing and instead referring only patients testing positive for P/LP variants. In our case, 24 535 patients would need to be referred to counseling before testing, an impractical number given the costs and the shortage of genetic counselors.^37^ Of course, this approach will result in testing for some patients who do not actually meet criteria or who have already sought risk-mitigating treatment.^38^ However, the potential harms of testing low-risk individuals are negligible, especially when genetic testing is covered by insurance or is offered free-of-charge by a population health study. Furthermore, being FHS7 positive was associated with a 3-fold increase in the risk of being *BRCA1/2* positive for both sexes and a 44% increase in cancer risk among living female individuals, indicating that FHS7 is useful for indicating genetic risk even without counselor validation.

Reducing costs helps make the risk assessment process sustainable, which is necessary since FHS7-negative patients should be periodically reassessed to capture relevant family history changes before disease onset. We found that among patients who had both a personal history and a family history of a HBOC-related cancer in their EHR, more than one-half had a personal history diagnosis recorded first. Since hereditary risk would ideally be identified before the onset of disease, a simple questionnaire administered directly to patients every few years may allow earlier identification than relying on EHR family history assessments.

A direct questionnaire would also improve data reliability compared with using EHR family history data, which is often not comprehensive^39^ or does not include negative family history. Furthermore, more than two-thirds of the relevant EHR family history was recorded using free-text comments, with practitioners using free text at a higher rate than patients. Without natural language processing^40,41^ or the parsing methods we used, these data are lost for population health management purposes. Also, the low rate at which age of onset was recorded in EHR family history may lead to many at-risk patients being missed, because FHS7 uses age of onset. These factors may help explain why the sensitivity of EHR-derived FHS7 for identifying *BRCA1/2*-positive patients was relatively low, with only 38% of female *BRCA*1/2-positive and 20% of male *BRCA*1/2-positive individuals being FHS7 positive. These shortcomings, combined with our results showing that the survey identified many additional patients, suggest that a direct questionnaire can capture more at-risk patients while saving time for practitioners^42^ and reducing costs. Previous studies^43,44,45,46^ have also shown that patient-entered family history is reliable and more comprehensive than routinely collected family history. To reduce the burden on patients, the FHS7 questions can be ordered by descending frequency of affirmative responses and the questionnaire terminated after the first affirmative response, since only 1 affirmative response indicates referral for testing.

Finally, using a direct questionnaire rather than existing EHR family history data can make the risk assessment process more equitable, since EHR family history data collection is affected by sociodemographic factors.^47,48^ In our study, patients meeting EHR-derived FHS7 criteria were more likely to be White, older, and female; to live in wealthier neighborhoods; and to have private insurance and a higher level of health care utilization. Thus, using EHR-derived FHS7 is likely to perpetuate existing disparities and result in inequitable access to genetic testing. A direct questionnaire will not completely eliminate biases: previous work^49^ has shown that male sex and lower annual household income are associated with lower levels of family history knowledge, and in our survey female individuals were more likely to be FHS7 positive than male individuals. However, devoting resources specifically to providing the questionnaire to underserved patients should help. Ethnic and racial biases in EHR-derived FHS7 warrant further study because differences in the incidence rate of cancers^28^ may contribute to these biases.

Although not addressed by USPSTF^21^ or Nevada’s SB251,^20^ FHS7 appears to be useful for screening in both male and female individuals, because in our study FHS7-positive status was equally associated with *BRCA1/2*-positive status in both sexes. It is not particularly surprising that in our cohort analysis we failed to detect an association between male FHS7-positive status and cancer incidence, since *BRCA1/2*-positive male individuals are known to have a lower cancer risk than *BRCA1/2*-positive female individuals. Nevertheless, identified *BRCA1/2*-positive male individuals benefit from early screenings for cancers such as prostate and male breast cancer,^50^ and their female relatives may benefit from cascade screening.

Others^51,52,53^ have also reported population-level genetic screening programs using EHR family history to identify individuals with higher risk. However, we confirmed the usefulness of EHR family history for identifying patients with P/LP variants in a large sequenced population, which, to our knowledge, has not previously been performed. Some studies have used questionnaires alone or in combination with EHR data to assess risk, but study populations have generally been small^22,52,54^ or the questionnaires were offered to a select population.^38^ Thus, future work that began with iterative small-scale implementation tests (see Powell et al^55^) and ended with evaluating the effectiveness of an FHS7 questionnaire administered to patients across an entire health system would be valuable. Questionnaires similar to FHS7 may also be useful for screening for the other Centers for Disease Control and Prevention Tier 1 conditions (Lynch Syndrome^56^ and familial hypercholesterolemia).

### Limitations

Analysis limitations include the use of observational study designs, which were subject to self-selection biases. In particular, the rate of *BRCA1/2*-positive participants was likely higher among HNP enrollees than would be expected in the general population, and major differences in sociodemographics and health care utilization were observed between HNP participants and the general patient population. The 57.8% FHS7-positive rate among survey respondents and the 21.8% response rate also suggest self-selection among the surveyed population, and it should be noted that the survey FHS7 questions were preceded by other family history questions and, thus, were not equivalent to a stand-alone questionnaire. In addition, informative presence bias (resulting from the tendency of less healthy patients to have more data available in their EHRs)^57^ could influence our analysis of cancer incidence rates, despite efforts to adjust for health care utilization levels.

## Conclusions

In the cross-sectional analysis, we found a large gap between the number of patients meeting family history criteria and those being genetically tested for HBOC in accordance with USPSTF and state guidelines. We also found an association between family history criteria and genetic risk variants, highlighting the importance of improving the risk assessment process and demonstrating the utility of EHR family history for identifying patients who would benefit from genetic testing. Owing to the shortcomings in EHR family history, implementing a direct FHS7 questionnaire would likely identify even more patients with HBOC. This would enable more cascade screening of family members and more preventive measures to be taken, thus reducing cancer mortality and morbidity.

## Notes

Supplement 1. **eMethods.** Methods for extracting HBOC-related cancers from free-text comments in the family history table **eTable 1. **Stratification variables used to adjust baseline hazard for cause-specific hazard models **eTable 2.** Top 20 comment/code combinations in the EHR **eTable 3.** Retrospective cohorts study population demographics and other characteristics

Supplement 2.
Data Sharing Statement
