# Supplementary material for: Screening Familial Risk for Hereditary Breast and Ovarian Cancer
Source: JAMA Netw Open. 2024 Sep 25;7(9):e2435901. doi: 10.1001/jamanetworkopen.2024.35901 (PMC11425146; doi:10.1001/jamanetworkopen.2024.35901)
Supplement: Supplement 1. — eMethods. Methods for extracting HBOC-related cancers from free-text comments in the family history table eTable 1. Stratification variables used to adjust baseline hazard for cause-specific hazard models eTable 2. Top 20 comment/code combinations in the EHR eTable 3. Retrospective cohorts study population demographics and other characteristics [file jamanetwopen-e2435901-s001.pdf]

Supplemental Online Content

Kiser D, Elhanan G, Bolze A, et al. Screening familial risk for hereditary breast and ovarian cancer. *JAMA Netw Open*. 2024;7(9):e2435901. doi:10.1001/jamanetworkopen.2024.35901

eMethods. Methods for extracting HBOC-related cancers from free-text comments in the family history table

eTable 1. Stratification variables used to adjust baseline hazard for cause-specific hazard models

eTable 2. Top 20 comment/code combinations in the EHR

eTable 3. Retrospective cohorts study population demographics and other characteristics

This supplemental material has been provided by the authors to give readers additional information about their work.

© 2024 Kiser D et al. *JAMA Network Open.*

eMethods. Methods for extracting HBOC-related cancers from free-text comments in the family history table

SQL query logic for identifying HBOC-related conditions in free-text comments:

Breast cancer:

```
case when
        (code = “Cancer” and
          (comments like '%breast%' or
          comments like '%br ca%' or
          comments COLLATE Latin1_General_CS_AS = 'BrCa' or
          comments like '%breasst%' or
          comments like '%breat%' or
          comments like '%mastectomy%')) or
        (code = “Other” and
          (comments like '%breast ca%' or
          (comments like '%cancer%' and comments like '%breast%') or
          comments like '%breat ca%' or
          comments like '%breast/lung ca%' or
          comments COLLATE Latin1_General_CS_AS = 'BR CA' or
          comments COLLATE Latin1_General_CS_AS = 'BrCA' or
          comments COLLATE Latin1_General_CS_AS = 'BrCa'))
        then 1 else 0
        end as comments_breast
```

Ovarian Cancer:

```
case when
        (code = “Cancer” and
          comments like '%ovar%') or
        (code = “Other” and
          (comments like '%ovarian ca%'))
        then 1 else 0
        end as comments_ovarian
```

Fallopian Tubal Cancer:

```
case when
        (code = “Cancer” and
          (comments like '%fallop%' or
          comments like '%falop%' or
          comments like '%tubal%'))
        then 1 else 0
        end as comments_fallop
```

Prostate Cancer:

```
case when
        (code = “Cancer” and
          (comments like '%prosta%' or
          comments like '%prossta%')) or
        (code = “Other” and
          (comments like '%prostate ca%' or
          comments like '%ca prostate%' or
          comments like '%ca of prostate%' or
          comments like '%ca (prostate%'))
        then 1 else 0
```

© 2024 Kiser D et al. *JAMA Network Open.*

```
            end as comments_prostate
```

Colorectal Cancer:

```
case when
            (code = "Cancer" and
              ((comments like '%colo%' and
                comments not like '%pol%' and
                comments not like '%colog%') or
              comments like '%rectal%' or
              comments like '%rectum%' or
              comments like '%bowel%')) or
            (code = "Other" and
              (comments like '%colon ca%' or
              comments COLLATE Latin1_General_CS_AS = 'olon cancer' or
              comments like '%rectal ca%')) or
            (code = "GI disease" and
              (comments like '%colon ca%' or
              comments like '%colon, ca%'))
            then 1 else 0
            end as comments_colorect
```

Melanoma:

```
case when
            (code = "Cancer" and
              (comments like '%melanoma%' and
                comments not like '%nonmelanoma%' and
                comments not like '%non melanoma%' and
                comments not like '%non-melanoma%' and
                comments not like '%non -melanoma%' and
                comments not like '%no melanoma%' and
                comments not like '%not melanoma%' and
                comments not like '%not-melanoma%' and
                comments not like '%non-malignant melanoma%')) or
            (code = "Other" and
              (comments like '%melanoma%' and
                comments not like '%non melanoma%' and
                comments not like '%non-melanoma%'))
            then 1 else 0
            end as comments_melanoma
```

Pancreatic Cancer:

```
case when
            (code = "Cancer" and
              comments like '%panc%') or
            (code = "Other" and
              (comments like '%pancreatic ca%' or
              comments like '%pancreas ca%' or
              comments like '%pancreatic acncer%' or
              comments like '%cancer pancreati%' or
              comments like '%cancer, panc%'))
            then 1 else 0
            end as comments_pancreatic
```

© 2024 Kiser D et al. *JAMA Network Open.*

Peritoneal Cancer:

```
case when
          (code = “Cancer” and
            (comments like '%perit%' and
              comments not like '%peritoni%' and
              comments not like '%retroperit%') or
            comments like '%pertioneal%' or
            comments like '%paritoneal%' or
            comments like '%paritenum%') or
          (code = “Other” and
            comments like '%peritoneal ca%')
          then 1 else 0
          end as comments_peritoneal
```

Records identified using free-text in the comments were excluded on a case-by-case basis if they included the following strings indicating negation:

"neg", "no ", "non", "not "

All records were excluded if they included the following strings indicating uncertainty (case-insensitive):

"?", "poss", "maybe", "i think", "not sure", "unsure", "not confident", "not to sure", "not completely sure", "does not know", "not 100% sure", "probable", "cannot recall", "suspected", "uncertain", "unclear"

A random sample of 2,000 records from unique patients was taken to assess the error rate in using free-text comments to identify HBOC-related cancers in the FHx. 7 errors were identified, for an error rate of 0.35%. 4 errors were due to undetected uncertainty in the comment, and 3 errors were due to comments referencing procedures or other conditions.

© 2024 Kiser D et al. *JAMA Network Open.*

eTable 1. Stratification variables used to adjust baseline hazard for cause-specific hazard models.

Continuous variables were grouped based on even increments; in the cases of census tract median household income and number of PHx/FHx assessments, the highest grouping was chosen such that it would have adequate numbers. Patients who were missing data for race/ethnicity or who were unable to be mapped to a census tract median household income were categorized as "unknown," patients who were missing language data were grouped with English speakers ("English or not indicated"), and patients who had no insurance information because they had not been billed were categorized as "no billing."

Stratification variables	Levels
**Age, years**	
	18-29
	30-39
	40-49
	50-59
	60-69
	70-79
**Race**	
	Asian
	Black
	Native American
	Pacific Islander
	White
	multiracial
	other
	unknown
**Ethnicity**	
	Hispanic
	non-Hispanic
	unknown
**Language**	
	non-English speaking
	English or not indicated
**Census tract median household income, $1k**	
	<50
	≥ 50 and < 100
	≥ 100
	unknown
**PHx/FHx updates, No.**	
	1-5
	6-10
	11-15
	16-20
	21-25
	>25

© 2024 Kiser D et al. *JAMA Network Open.*

eTable 1 (continued) – Stratification variables used to adjust baseline hazard for cause-specific hazard models. Continuous variables were grouped based on even increments; in the cases of census tract median household income and number of PHx/FHx assessments, the highest grouping was chosen such that it would have adequate numbers. Patients who were missing data for race/ethnicity or who were unable to be mapped to a census tract median household income were categorized as "unknown," patients who were missing language data were grouped with English speakers ("English or not indicated"), and patients who had no insurance information because they had not been billed were categorized as "no billing."

Stratification variables	Levels
**Monetization, deciles**	
	≤ \$655.00 (bottom 2 deciles)
	> \$655.00 and ≤ \$1,411.00 (3rd decile)
	> \$1,411.00 and ≤ \$2,798.00 (4th decile)
	> \$2,798.00 and ≤ \$5,250.26 (5th decile)
	> \$5,250.26 and ≤ \$10,273.25 (6th decile)
	> \$10,273.25 and ≤ \$18245.47 (7th decile)
	> \$18245.47 and ≤ \$31,730.25 (8th decile)
	> \$31,730.25 and ≤ \$63,124.76 (9th decile)
	> \$63,124.76 (10th decile)
**health insurance category**	
	private (never used Medicaid)
	Medicare (never used private, Medicaid)
	Medicaid (ever used)
	self-pay only
	other
	no billing
**prior colorectal cancer**	
	yes
	no
**prior melanoma**	
	yes
	no
**prior peritoneal cancer**	
	yes
	no
**prior pancreatic cancer (female model only)**	
	yes
	no

© 2024 Kiser D et al. *JAMA Network Open.*

eTable 2. Top 20 comment/code combinations in the EHR.

Frequency is calculated prior to removing historical records, so the total number of FHx records with HBOC-related cancers in the comments is much larger than reported in the main results. The top 20 comment/code combinations represent 78.3% of the 1,887,669 total records identified via comments (rather than via discrete codes specific to a cancer type). Thus, most comments clearly indicate the type of HBOC-related cancer.

rank	code	comments	No. (%) of comments
1	Cancer	breast	500,147 (26.5%)
2	Cancer	colon	196,438 (10.4%)
3	Cancer	prostate	168,921 (8.9%)
4	Cancer	breast cancer	145,113 (7.7%)
5	Cancer	melanoma	71,872 (3.8%)
6	Cancer	colon cancer	67,649 (3.6%)
7	Cancer	pancreatic	63,206 (3.3%)
8	Cancer	ovarian	58,783 (3.1%)
9	Cancer	prostate cancer	40,523 (2.1%)
10	Cancer	breast ca	31,010 (1.6%)
11	Cancer	pancreatic cancer	22,672 (1.2%)
12	Cancer	breast	20,423 (1.1%)
13	Cancer	ovarian cancer	17,198 (0.9%)
14	Cancer	colon ca	15,977 (0.8%)
15	Cancer	prostate ca	12,924 (0.7%)
16	Cancer	pancreas	12,759 (0.7%)
17	Cancer	breast cancer	12,181 (0.6%)
18	Cancer	colon	9,328 (0.5%)
19	Cancer	colon cancer	6,500 (0.3%)
20	Cancer	prostate	5,225 (0.3%)

© 2024 Kiser D et al. *JAMA Network Open.*

eTable 3. Retrospective cohorts study population demographics and other characteristics.

	Females		Males	
	FHS7-	FHS7+	FHS7-	FHS7+
**No.**	125,488	6,134	112,645	2,337
**under the age of 50, No. (%)**	69,312 (55.2)	2,029 (33.1)	59,975 (53.2)	704 (30.1)
**sex, No. (%)**				
female	125,488 (100.0)	6,134 (100.0)	0 (0.0)	0 (0.0)
male	0 (0.0)	0 (0.0)	112,645 (100.0)	2,337 (100.0)
**known race, No. (%)**	123,007 (98.0)	6,026 (98.2)	110,293 (97.9)	2,287 (97.9)
**race, No. (% of known race)**				
Asian	4,875 (4.0)	196 (3.3)	2,725 (2.5)	61 (2.7)
Black	2,564 (2.1)	92 (1.5)	3,092 (2.8)	35 (1.5)
Native American	2,259 (1.8)	34 (0.6)	1,501 (1.4)	11 (0.5)
Pacific Islander	706 (0.6)	16 (0.3)	503 (0.5)	6 (0.3)
White	100,053 (81.3)	5,446 (90.4)	91,078 (82.6)	2,084 (91.1)
multiracial	1,581 (1.3)	38 (0.6)	1,233 (1.1)	9 (0.4)
other	10,969 (8.9)	204 (3.4)	10,161 (9.2)	81 (3.5)
**Hispanic ethnicity, No. (%)**	21,925 (17.5)	476 (7.8)	17,351 (15.4)	142 (6.1)
**non-English speaking, No. (%)**	5,039 (4.0)	56 (0.9)	2,505 (2.2)	12 (0.5)
**known census tract, No. (%)**	125,471 (100.0)	6,133 (100.0)	112,631 (100.0)	2,337 (100.0)
**median household income of census tract of residence, No. (% of known census tract)**				
<$50k	24,298 (19.4)	775 (12.6)	22,694 (20.1)	320 (13.7)
$50-99k	79,991 (63.8)	3,950 (64.4)	70,879 (62.9)	1,483 (63.5)
≥$100k	21,182 (16.9)	1,408 (23.0)	19,058 (16.9)	534 (22.8)
**patients with charges, No. (%)**	119,107 (94.9)	5,971 (97.3)	105,172 (93.4)	2,284 (97.7)
**health insurance category, No. (% of patients with charges)**				
private (no Medicaid)	69,135 (58.0)	3,969 (66.5)	63,008 (59.9)	1,626 (71.2)
Medicare (no private/Medicaid)	14,616 (12.3)	1,120 (18.8)	12,845 (12.2)	392 (17.2)
Medicaid (ever used)	26,382 (22.1)	691 (11.6)	16,123 (15.3)	194 (8.5)
self-pay only	4,315 (3.6)	47 (0.8)	6,595 (6.3)	20 (0.9)
other	4,659 (3.9)	144 (2.4)	6,601 (6.3)	52 (2.3)
**health care utilization, median (IQR)**				
PHx/FHx updates, No.	6 (3, 13)	13 (7, 23)	4 (2, 9)	10 (5, 17)
monetization	10.0 (1.9, 32.8)	15.9 (4.5, 46.6)	5.7 (1.1, 25.5)	11.1 (2.9, 38.1)

[a] charges to a patient's account

© 2024 Kiser D et al. *JAMA Network Open.*

eTable 3 (continued) – Retrospective cohorts study population demographics and other characteristics.

	Females		Males	
	**FHS7-**	**FHS7+**	**FHS7-**	**FHS7+**
**personal history of cancer, No. (%)**				
any cancer	1,268 (1.0)	143 (2.3)	1,245 (1.1)	78 (3.3)
breast	0 (0.0)	0 (0.0)	0 (0.0)	0 (0.0)
colorectal	492 (0.4)	59 (1.0)	523 (0.5)	31 (1.3)
melanoma	715 (0.6)	80 (1.3)	727 (0.6)	50 (2.1)
ovarian	0 (0.0)	0 (0.0)	0 (0.0)	0 (0.0)
pancreatic	67 (0.1)	4 (0.1)	0 (0.0)	0 (0.0)
peritoneal	7 (0.0)	1 (0.0)	4 (0.0)	0 (0.0)
prostate	0 (0.0)	0 (0.0)	0 (0.0)	0 (0.0)
tubal	0 (0.0)	0 (0.0)	0 (0.0)	0 (0.0)
**prior knowledge of HBOC, No. (%)**				
diagnosis	48 (0.0)	23 (0.4)	8 (0.0)	1 (0.0)
HNP participant	0 (0.0)	0 (0.0)	0 (0.0)	0 (0.0)
no diagnosis/non-HNP	125,440 (100.0)	6,111 (99.6)	112,637 (100.0)	2,336 (100.0)
**family history records, No. (%)**				
HBOC-related FHx	8,469 (6.7)	6,134 (100.0)	4,161 (3.7)	2,337 (100.0)
FHS7+	0 (0.0)	6,134 (100.0)	0 (0.0)	2,337 (100.0)

[a] charges to a patient's account

© 2024 Kiser D et al. *JAMA Network Open.*
