# Supplementary material for: Screening Familial Risk for Hereditary Breast and Ovarian Cancer
Source: JAMA Netw Open. 2024 Sep 25;7(9):e2435901. doi: 10.1001/jamanetworkopen.2024.35901 (PMC11425146; doi:10.1001/jamanetworkopen.2024.35901)
Supplement: Supplement 2. — Data Sharing Statement [file jamanetwopen-e2435901-s002.pdf]

Data Sharing Statement

Kiser. Screening Familial Risk for Hereditary Breast and Ovarian Cancer. *JAMA Netw Open*. Published September 25, 2024. doi:10.1001/jamanetworkopen.2024.35901

Data
Data available: Yes
Data types: Deidentified participant data
How to access data: Comma-separated-values files containing the data used to create Tables 1, 3, and 4 of the manuscript can be downloaded from Dryad by following this link: https://doi.org/10.5061/dryad.gf1vhhmxr. datashare1.csv contains records for 835,727 patients indicating their FHS7 status according to their EHRs. For 1,527 survey respondents, the records also indicate their FHS7 status according to their survey responses. datashare2.csv contains records for each of 37,996 HNP participants indicating FHS7 status, sex, and genes with P/LP variants. Due to privacy concerns, a small number of patients with sex recorded as non-binary or transgender were excluded. Data used to create Table 2 and to train the cause-specific hazard models are subject to HIPAA and other privacy and compliance restrictions. Requests for these and other data may be addressed to Joe Grzymski (at jgrzymski@med.unr.edu) or Craig Kugler (at ckugler@med.unr.edu).
When available: With publication

Supporting Documents
Document types: Statistical/analytic code
How to access documents: The R code used for producing Tables 1, 3 and 4 can be downloaded from Dryad at https://doi.org/10.5061/dryad.gf1vhhmxr just like the individual de-identified data.
When available: With publication

Additional Information
Who can access the data: The data posted on Dryad are available to anyone for research and educational reuse. Data used to create Table 2 and to train the cause-specific hazard models are available upon request. These data (and other sensitive data) are available to qualified researchers upon reasonable request and with permission from the Center for Genomic Medicine. The HNP encourages collaboration with scientific researchers on an individual basis. Examples of restrictions that will be considered in requests to data access include but are not limited to: 1. Whether the request comes from an academic institution in good standing that will collaborate with our team to protect the privacy of the participants and the security of the data requested 2. Type and amount of data requested. 3. Feasibility of the research suggested. 4. Amount of resource allocation to support the collaboration.
Types of analyses: Data available in datashare1.csv can be used to replicate Table 1 in the main manuscript, and data available in datashare2.csv can be used to replicated Tables 3 and 4 in the main manuscript. Other purposes are also permitted.
Mechanisms of data availability: Data on Dryad can be freely downloaded. Other data requires investigator support, approval of the proposal, and possibly a signed data access agreement, depending on the sensitivity of the data requested.
